# Evaluation of the G145R Mutant of the Hepatitis B Virus as a Minor Strain in Mother-to-Child Transmission

**DOI:** 10.1371/journal.pone.0165674

**Published:** 2016-11-03

**Authors:** Haruki Komatsu, Ayano Inui, Shuichiro Umetsu, Tomoyuki Tsunoda, Tsuyoshi Sogo, Yasuhiro Konishi, Tomoo Fujisawa

**Affiliations:** 1 Department of Pediatrics, Toho University, Sakura Medical Center, Chiba, Japan; 2 Department of Pediatric Hepatology and Gastroenterology, Eastern Yokohama Hospital, Kanagawa, Japan; 3 Department of Obstetrics & Gynecology, Eastern Yokohama Hospital, Kanagawa, Japan; Defence Research Laboratory, INDIA

## Abstract

The role of the hepatitis B virus (HBV) mutant G145R, with a single change in amino acid 145 of the surface protein, as a minor population remains unknown in mother-to-child transmission. The minor strain as well as the major strain of the G145R mutant were evaluated in three cohorts using a locked nucleic acid probe-based real-time PCR. The breakthrough cohort consisted of children who were born to HBV carrier mothers and became HBV carriers despite immnoprophylaxis (n = 25). The control cohort consisted of HBV carriers who had no history of receiving the hepatitis B vaccine, hepatitis B immunoglobulin or antiviral treatment (n = 126). The pregnant cohort comprised pregnant women with chronic HBV infection (n = 31). In the breakthrough cohort, 6 showed positive PCR results (major, 2; minor, 4). In the control cohort, 13 showed positive PCR results (major, 0; minor, 13). HBeAg-positive patients were prone to have the G145R mutant as a minor population. Deep sequencing was performed in a total of 32 children (PCR positive, n = 13; negative, n = 19). In the breakthrough cohort, the frequency of the G145R mutant ranged from 0.54% to 6.58%. In the control cohort, the frequency of the G145R mutant ranged from 0.42% to 4.1%. Of the 31 pregnant women, 4 showed positive PCR results (major, n = 0; minor, n = 4). All of the pregnant women were positive for HBeAg and showed a high viral load. Three babies born to 3 pregnant women with the G145R mutant were evaluated. After the completion of immunoprophylaxis, 2 infants became negative for HBsAg. The remaining infant became negative for HBsAg after the first dose of HB vaccine. G145R was detected in one-fourth of the children with immunoprophylaxis failure. However, the pre-existence of the G145R mutant as a minor population in pregnant women does not always cause breakthrough infection in infants.

## Introduction

Although the hepatitis B (HB) vaccine, which prevents the transmission of hepatitis B virus (HBV), was introduced into routine immunization programs in 183 countries by 2013 [1], HBV infection remains a major global health problem. On the basis of data from the World Health Organization, an estimated 240 million people suffer from chronic HBV infection [1]. People infected with chronic HBV infection have a high risk of developing liver cirrhosis and hepatocellular carcinoma. HBV is transmitted from person to person through blood or bodily fluids. Mother-to-child transmission (MTCT) is one of the main transmission routes, especially in East Asia. To prevent mother-to-child transmission, a series of 3 doses of HB vaccine is administered to all newborns. In intermediate-income and high-income countries, a series of 3 doses of HB vaccine plus hepatitis B immunoglobulin (HBIG) combined with a universal maternal screening program are administered to newborn babies born to HBV carrier mothers. The protection rate of MTCT with the HB vaccine plus HBIG and vaccine alone was reported to be 94% and 75%, respectively [2]. Although immunoprophylaxis with the combination of HB vaccine and HBIG is highly effective for the prevention of MTCT, 5% to 10% of infants born to HBeAg/HBsAg-positive mothers become infected despite adequate immunoprophylaxis [3–7]. Apart from improper immunization, a high level of maternal viraemia is considered to be the main cause of immunoprophylaxis failure [5, 6, 8–12]. In addition to a maternal high viral load, vaccine escape mutants (VEMs) can play a crucial role in immunoprophylaxis failure in MTCT [13–17]. In the post universal vaccination era, the control of VEMs is one of the unresolved problems in HBV eradication. VEMs are rarely detectable in HBV carrier mothers, and infants are therefore affected by MTCT due to the emergence of VEMs [14, 15, 18–22]. It is hypothesized that VEMs are minor variants that are selected under pressure due to the HB vaccine and/or HBIG; these strains therefore become predominant in the viral population and contribute to immunoprophylaxis failure [14, 15]. Thus, evaluation of the VEMs as minor strains is indispensable for clarifying the mechanism of immunoprophylaxis failure in MTCT. Among the VEMs, the mutant G145R, with a single mutation of amino acid 145 of the surface protein of HBV, which is located in the “a” determinant region (aa124-147), is well known as the most virulent mutant in breakthrough infections [13, 15, 17]. However, it is difficult to detect the minor strain of G145R in HBV carriers by Sanger sequencing and conventional real-time PCR. A locked nucleic acid (LNA) is a nucleic acid analogue containing a methylene bridge that connects the 2’-oxygen of ribose with the 4’-carbon [23]. The introduction of LNA into the DNA oligomer improves the hybridization affinity for complementary sequences, and the LNA-based probe shows strong mismatch discriminatory power [24–26]. In this study, the G145R mutant as a minor strain as well as a major strain was detected in chronically infected children with HBV due to breakthrough infection using LNA-based probe real-time PCR. Moreover, the sequence of the HBV surface region was also evaluated using deep sequencing. Finally, a prospective study was conducted to clarify whether immunoprophylaxis failure could occur in infants who were born to HBV carrier mothers with the G145R mutant existing as a minor population.

## Patients and Methods

### Patients

From 2007 to 2015, patients with chronic HBV infection who visited the Department of Pediatrics of the Eastern Yokohama Hospital were enrolled in this study. Patients who had a history of receiving antiviral treatment, such as interferon and nucleos(t)ide analogues (NAs), were excluded. Moreover, patients who were positive for the hepatitis C virus (HCV) antibody and those who had another potential cause for chronic liver diseases (autoimmune hepatitis, primary sclerosing cholangitis, Wilson’s disease and non-alcoholic fatty liver disease) were also excluded from the study. Because the prevalence of anti-HDV is extremely low (0.6%) in Japan [27], anti-HDV was not measured in this study.

### Pregnant women and infants

In addition, pregnant women who were positive for HBsAg at universal antepartum screening performed in the second trimester of pregnancy at the Department of Gynecology and Obstetrics of the Yokohama Eastern Hospital between 2007 and 2014 were enrolled in this study. All of the pregnant women were negative for anti-HCV, anti-human immunodeficiency virus and anti-human T-cell leukaemia virus type 1 antibodies. Serum samples from HBsAg-positive pregnant women for this study were collected in the third trimester of pregnancy. According to the protocol of Yokohama Eastern Hospital, all infants born to HBsAg-positive mothers received HBIG (200 IU, Dried HB globulin-Nichiyaku, Nihon Pharmaceutical Co. Ltd, Tokyo, Japan) within 12 hours after birth and 3 doses of HB vaccine (5 μg, Bimmugen, Chemo-Sero-Therapeutic Research Institute, Kumamoto, Japan) within 1 week after birth and at 1 month and 5 months after birth. Serum HBsAg and anti-HBs were measured in infants born to HBV carrier mothers at 6 months after birth. When an infant was negative for serum HBsAg at 6 months after birth, the prevention of MTCT was considered to be successful.

The hepatitis B serologic viral markers (HBsAg, HBeAg, anti-HBe, and anti-HBs) were tested using a chemiluminescence enzyme immunoassay (EIA) (Lumipulse, FUJIREBO INC, Tokyo, Japan). Written informed consent was obtained from all parents or legal guardians. This study was approved by the ethical committee of Eastern Yokohama Hospital and Toho University Sakura Medical Center (No.2014059) and performed in accordance with the ethical guidelines of the 1975 Declaration of Helsinki.

### DNA extraction, direct sequencing, plasmid construction and HBV DNA quantification

HBV DNA was extracted from 200 μL of serum using a QIAamp DNA Blood Mini kit (QIAGEN, Hilden, Germany). The extracted DNA was dissolved in 100 μL of elution buffer. The surface regions of the HBV genome were amplified by nested PCR using 2 primer pairs that encompassed *a* determinant region, which was positioned between amino acids 124 and 147, as previously described [28]. Direct sequencing analysis was performed using the second-round PCR product (product size: 432 bp). To validate the LNA-based probe real-time PCR, wild-type and G145R mutant type (nucleotide position at 587: G →A) PCR products of the surface region (432bp) were purified and cloned into the pCR4 TOPO vector (Invitrogen, Carlsbad, CA) and transformed into TOP 10 One Shot *Escherichia coli* bacteria (Invitrogen). The recombinant plasmid DNA was purified using QuickLyse Miniprep (QIAGEN), and the concentration of HBV DNA was determined using a spectrophotometer. Nucleotide positions were designated on the basis of nucleotide sequences from genotype C (GenBank/EMBL accession number AB300361). Serum HBV DNA levels without discrimination between wild-type and mutant types were determined using a COBAS TaqMan HBV DNA test (Roche Diagnostics, Tokyo, Japan). Genotyping of HBV was determined with the PCR-Invader assay [29]

### LNA-based probe real-time PCR

The two sets of paired primers used for real-time PCR were as follows: forward 1: CCATGCAAGACCTGCA (nt 512–527), reverse 1: GATGATGGGATGGGAATACA (nt 599–618); (amplicon size: 111 bp), forward 2: CTCTTGTTGCTGTACAAAACC (nt 559–579), reverse 2: AGGCCCACTCCCATAG (nt 638–653); (amplicon size 99 bp). The following LNA-based probes (nt 582–591) designed for discrimination between the G145R mutant (nucleotide position at 587: G→A) and wild-type were used for real-time PCR: Probe-G (wild-type sequence); 5’-FAM-C**G**G**ACG**GAAA-IBFQ-3’, Probe-A (mutant-type sequence); 5’-HEX-**CG**G**ACA**G**A**AA-IBFQ-3’ (Integrated DNA Technologies, Coralville, Iowa). LNA nucleotides are given in bold and underlined letters. PCR was performed in a 25-μL reaction mixture containing 12.5 μL TaqMan Universal PCR master mix (Applied Biosystems, Foster City, CA) with 0.2 μM primers, 0.1 μM probes, and 5 μL extracted DNA. The PCR program consisted of an initial pre-cycle incubation at 50°C for 2 min and 95°C for 10 min, followed by 40 cycles of 95°C for 15 s and 60°C for 1 min. The PCR assay was performed in a MX3000P (Agilent Technologies, Santa Clara, CA), and the results were analysed with MxPro software (version 3.0). The lower detection limit was >1,000 copies/mL. All assays were carried out in triplicate with negative control samples. The wild-type and G145R mutant-type DNA extracted from serum were quantified according to the recombinant plasmid wild-type controls and the G145R mutant-type (nucleotide position at 587: G→A) controls, respectively.

### Ultra-deep pyrosequencing

The surface region of the HBV genome was amplified (432 bp) by nested PCR as described previously [28]. The outer primers were integrated with multiple identifiers and 454 specific adaptors. All samples were amplified with a proofreading enzyme (high-fidelity enzyme; PrimeSTAR MAX DNA polymerase, TaKaRa Bio) in PCR. The PCR amplicons were purified using AMPure XP (Beckman Coulter, Danvers, MA) and quantified using MulTiNA (Shimazu, Kyoto, Japan). The first round of PCR was performed in 35 cycles (denaturation at 98°C for 10 s, annealing at 55°C for 5 s, and extension at 72°C for 30 s), and the second PCR round was performed in 25 cycles under the same conditions as the first round. The PCR amplicons of each patient were pooled and subjected to ultra-deep pyrosequencing, carried out with a Roche GS junior sequencer. Sequence reads were aligned with the HBV reference sequence of HBV genotype C (GenBank/EMBL accession number AB300361) using the alignment tool BWA (0.7.6) (https://sourceforge.net/projects/bio-bwa/files). The analysis of read depth and base count was displayed using GATK (https://www.broadinstitute.org/gatk/download). An HBV recombinant plasmid clone of known sequence was amplified in triplicate, and ultra-deep sequencing was performed to check for technical errors. The median coverage for three control experiments was 6,757 (range: 5,803 to 8,438). The rate of technical errors ranged from 0.20% to 0.24% (mismatches) and from 0.15% to 0.39% (inserts/deletions). Therefore, the accuracy of ultra-deep sequencing in this platform for detecting low-level viral mutations was considered to be greater than 0.4%. Moreover, 10 or more of the nucleotide mutation counts were considered to be valid in preventing technical error.

### Statistics

Univariable tests of association for categorical patient characteristics were performed with Pearson’s chi-squared test if each of a table’s cell count was at least 5 and with Fisher’s exact test otherwise. For continuous variables, a Mann-Whitney-Wilcoxon rank-sum test was used. For multivariable tests of association, logistic regression was used. For all statistical summarizations and analyses, Stata MP software (version 14.1, StataCorp LP, College Station, TX) was used. A p value of 0.05 or less was considered to indicate statistical significance for all tests.

## Results

### Patients

One hundred fifty-one patients (M/F = 64/87, age: median 15 years) with chronic HBV infection were enrolled in this study. Of the 151 patients, 119 were enrolled in our previous study [28]. The subjects were divided into two cohorts. One was the control cohort comprising patients who had no history of receiving HB vaccines or HBIG. The other was the breakthrough cohort comprising patients born to HBV carrier mothers who had breakthrough infection despite immunoprophylaxis with a series of 3 doses of HB vaccine plus HBIG. Of the 151 patients, 126 (M/F = 53/73, age: median 17 years) were classified as the control cohort. The remaining 25 patients (M/F = 11/14, age: median 5 years) were classified as the breakthrough cohort. The characteristics of 126 patients who belonged to the control cohort are shown in Table 1. Of these 126 patients, 75 (60%) were positive for HBeAg, and the vast majority (87%) showed HBV genotype C. Although approximately half (54%) of the subjects were infected through MTCT, 45 (36%) were infected through unknown source transmissions. The characteristics of the 25 patients who belonged to the breakthrough cohort are shown in Table 2. These patients were younger than the control patients. Of these 25 patients, 22 (88%) were positive for HBeAg, and the median serum HBV DNA level was 8.5 log copies/mL.

**Table 1:** Characteristics of patients who had no history of receiving HB vaccine or HBIG (control cohort).

	All patients (n = 126)	LNA PCR for G145R		Univariate	Multivariate
		Positive (n = 13)	Negative (n = 113)	P value	P value
Age, median, year	17 (1–50)	13	17	0.44	0.7
Gender, male (%)	53 (42)	6 (47)	47 (42)	0.75	0.82
HBV DNA levels, median, log copies/mL	5.3 (2.1–9.0)	6.3	5.3	0.17	0.66
HBeAg, positive (%)	75 (60)	11 (85)	64 (57)	0.05	0.12
HBV genotype (%)				*1	*0.87
A	1 (1)	0	1 (1)		
B	15 (12)	1 (8)	14 (12)		
C	110 (87)	12 (92)	98 (87)		
Route of transmission (%)				**0.24	**0.38
Mother-to-child	68 (54)	9 (69)	59 (52)		
Father-to-child	11 (5)	0	11 (10)		
Grandparent-to-child	2 (2)	0	2 (2)		
Unknown	45 (36)	4 (31)	41 (36)		

* Genotype C vs. non-genotype C

** Mother-to-child transmission vs. non-mother-to-child transmission

**Table 2:** Children born to HBV carriers in whom breakthrough infection occurs despite of immunoprophylactic treatment (breakthrough cohort).

	All patients (n = 25)	LNA PCR for G145R		Univariate
		Positive (n = 6)	Negative (n = 19)	P value
Age, median, year	5 (1–13)	4	5	0.58
Gender, male (%)	11 (44)	2 (33)	9 (47)	0.41
HBV DNA levels, median, log copies/mL	8.5 (2.5–9)	7.7	8.6	0.97
HBeAg, positive (%)	22 (88)	6 (100)	16 (84)	0.75
HBV genotype (%)				
B	1 (4)	1 (17)	0	
C	24 (96)	5 (83)	19 (100)	

### Direct sequencing

Direct sequencing was performed in 126 patients (control cohort) and 25 patients with breakthrough infection (breakthrough cohort). None of patients belonging to the control cohort showed evidence of the G145R mutant. Of the 25 patients belonging to the breakthrough cohort, however, 2 (8%) had the G145R mutant (GGA→AGA) and one (4%) had the G145K mutant (GGA→AAA).

### LNA-based probe real-time PCR

To evaluate the specificity of the LNA based probe, wild-type and G145R mutant type (nucleotide position at 587: G→A) recombinant plasmid DNA were used for the LNA-based probe real-time PCR. Because high levels of viral DNA caused false positive results in our previous study [28], we initially evaluated the specificity of the LNA probes using high levels of plasmid DNA. When wild-type plasmid DNA (2×10^11^ copies/mL) was applied to real-time PCR, the G145R mutant was undetectable. Similarly, when the G145R mutant-type plasmid DNA (5×10^10^ copies/mL) was applied in real-time PCR, the wild-type was also undetectable. Although the specificity of the LNA-based probe real-time PCR was evaluated in various plasmid DNA levels ranging from 10^11^ copies/mL to 10^3^ copies/mL, the LNA probes showed neither false negative results nor false positive results. Moreover, we confirmed that the LNA-based probe real-time PCR could detect 1,000 copies/mL or more in both wild-type and G145R mutant-type plasmid DNA. Then, we evaluated the discrimination ability of the LNA-base probe between wild-type and G145R plasmid DNA in the mixture of wild-type and G145R mutant DNA. The wild-type and G145R mutant plasmids were mixed in the 25-μL PCR mixture as follows: (A) wild:mutant = 2:1, the wild-type [2×10^8^ copies/mL] and the mutant-type [1×10^8^copies/mL] were mixed, (B) wild:mutant = 20:1, the wild-type [2×10^8^ copies/mL] and the mutant-type [1×10^7^copies/mL] were mixed, (C) wild:mutant = 200:1, the wild-type [2×10^8^ copies/mL] and the mutant-type [1×10^6^ copies/mL] were mixed, and (D) wild:mutant = 2,000:1, the wild-type [2×10^8^ copies/mL] and the mutant-type [1×10^5^ copies/mL] were mixed. The LNA-based probe could detect the mutant-type plasmid DNA as a minor population in the majority of wild-type plasmid DNA (wild:mutant = 2:1, 20:1, and 200:1). However, when the mutant/wild ratio was 1:2,000 (0.05%), this LNA-based probe failed to detect mutant plasmid DNA as a minor population in the majority of wild-type plasmid DNA. These findings suggested that the lower limitation of the mutant/wild ratio was 1/200 (0.5%).

### Positive rate of the LNA-based probe

Of the 126 patients in the control cohort, 13 (10.3%) showed positive results for LNA-based probe real-time PCR (Table 1). All of the 13 patients had the G145R mutant as a minor population. A pair of siblings (Nos. 143 and 144 in Table 3) and a mother and child pair (Nos. 107 and 108 in Table 3) were observed in the control cohort. Table 1 shows the predictive factors for the detection of the G145R mutant. Age, gender, HBV DNA level, HBeAg, HBV genotype (genotype C), and transmission route (MTCT) were evaluated for association with the detection of G145R. Univariate analysis showed that the positivity of HBeAg had a close association with the detection of the G145R mutant (p = 0.05). However, multivariate analysis showed that the positivity of HBeAg was not significantly associated with detection of the G145R mutant (p = 0.12).

**Table 3:** Characteristics of patients who were positive for the LNA-based probe real-time PCR assay.

Subjects	Patient No.	Gender	Age	HBeAg	HBV genotype	Serum HBV DNA levels	*PCR cloning mutant no./wild no. (%)	Quantitative PCR	Deep sequencing
								Mutant/Wild HBV DNA ratio, %	Mutation frequency, % nucleotide position at 587: G→A
Breakthrough cohort (N = 6)	35	M	6	Pos	C	6.9	ND	6	0.64
	48	F	4	Pos	B	8.1	ND	5.7	0.54
	52	F	1	Pos	C	6.9	ND	No detection of wild-type	Major
	81	F	9	Pos	C	7.2	5/15 clones (33)	30	6.58
	136	F	3	Pos	C	8.8	ND	More than 100	Major
	139	M	4	Pos	C	9	ND	3	0.88
Control cohort(N = 13)	9	M	14	Pos	C	4.9	ND	10	1.75
	33	F	8	Pos	C	6.1	0/32 clones	3.3	1.82
	37	M	12	Pos	C	6.9	2/12 clones (17)	5	ND
	38	F	10	Neg	C	4.3	0/12 clones	13	(0.25)**
	71	F	33	Pos	C	9	ND	4.3	1.11
	92	M	15	Neg	C	3.3	2/13 clones (15)	50	ND
	97	M	13	Pos	C	4.1	1/24 clones (4)	5	ND
	106	F	40	Pos	C	8.8	1/15 clones (7)	10	ND
	107	F	43	Pos	C	5.2	2/14 clones (14)	15	ND
	108	M	9	Pos	C	8.8	ND	2	0.58
	143	F	10	Pos	C	7.4	1/14 clones (7)	7.5	ND
	144	M	9	Pos	C	7.2	ND	2	0.42
	151	F	42	Pos	B	6.3	0/30 clones	5	4.1

*PCR cloning was performed in our previous study (ref.No.28).

** Mutation frequency was not considered to be valid. ND; not done

In contrast to the control cohort, 6 (24%) of the 25 patients in the breakthrough cohort showed positive results for LNA-based probe real-time PCR (Table 2). Of these 6 patients, 2 had the G145R mutant as a major strain, which was consistent with the results of direct sequencing. The remaining 4 patients had the G145R mutant as a minor strain. The positive rate of the breakthrough cohort was approximately 2-fold higher than that of the control cohort. Although immunoprophylaxis failure was related to the detection of the G145R mutant, there was no significant difference in the positive rate from real-time PCR between the two cohorts (OR value = 2.74, 95% CI: 0.93–8.10, P = 0.06). Univariate analysis showed that there was no predictive factor for the detection of G145R in the breakthrough cohort (Table 2).

The characteristics of patients who showed positive results for the LNA-based probe real-time PCR are shown in Table 3. In our previous study, the presence of the G145R mutant was evaluated by cloning the PCR product [28]. Of the 19 patients with positive results for the LNA-based probe real-time PCR in this study, 10 (breakthrough cohort; 1, control cohort; 9) were evaluated by cloning the PCR product in our previous study. Of these 10 patients, 7 (breakthrough cohort;1, control cohort; 6) were already confirmed to have the G145R mutant as a minor form in the previous study [28]. However, the remaining 3 patients were negative for the G145R mutant based on the cloning and sequencing of PCR products in our previous study. These findings suggested that the LNA-based probe real-time PCR was superior to the cloning technique in the detection of the G145R mutant and could distinguish the G145R mutant existing as a low percentage of the excessive wild-type viral population.

The quantifications of wild-type DNA and G145R mutant DNA were performed in the 19 patients who showed positive results for the LNA-based probe real-time PCR. The ratio of G145R mutant DNA to wild-type DNA (mutant/wild ratio) is shown in Table 3. Of the two patients who had the G145G mutant as a predominant strain (patient Nos. 52 and 136), one was negative for wild-type DNA, and the other showed a mutant/wild ratio of more than 100%. The remaining 17 patients had the G145R mutant as a minor population and the mutant/wild ratio ranged from 2% to 50%. As shown in Table 3, the mutant/wild ratio calculated by the LNA-based probe real-time PCR was consistent with the mutant/wild ratio calculated by the cloning technique.

### Ultra-deep sequencing

There were two objectives when we performed deep sequencing. One was the confirmation of the existence of the G145R mutant as a minor strain. The other was to evaluate the association between other VEMs as minor strains and breakthrough infections in MTCT. Deep sequencing was performed in all of the 25 patients who belonged to the breakthrough cohort and the 7 patients belonging to the control cohort who were positive for the LNA-based probe real-time PCR, but in whom the G145R mutant was not confirmed by cloning of a PCR product. The median coverage per sample was 8,768 reads (range; 3,434 to 18,523). Of the 25 patients in the breakthrough cohort, 6 showed that the frequency of the G145R mutation (nucleotide position at 587: G→A) was 0.4% or more (Table 3); all of these patients showed positive results for the LNA-based probe real-time PCR. The frequency of the G145R mutation ranged from 0.54% to 6.58% in 4 patients belonging to the breakthrough cohort with the G145R mutant as a minor strain. Of the 7 patients belonging to the control cohort, the frequency of the G145R mutation was 0.4% or more in all but one. The frequency of G145R ranged from 0.42% to 4.10%. In one patient, who was positive for the LNA-based probe real-time PCR, the frequency of the G145R mutation was 0.25%, which was not considered to be valid. In contrast to the LNA-based probe real-time PCR-positive patients, the frequency of the G145R mutation ranged from 0% to 0.0007% in 18 of 19 patients who were negative for the real-time PCR. Because the remaining patient had the G145K (GGA→AAA) mutant as a major strain, which was confirmed by direct sequencing, the frequency of the G to A change at nt.587 was 98.6%. These findings indicated that LNA-based probe real-time PCR could accurately detect the G145R mutant as a minor population. We retrospectively evaluated 4 of 6 HBV carrier mothers whose children were positive for the LNA-based probe real-time PCR in the breakthrough cohort (G145R major strain; No. 52 and No. 136, minor strain; No. 35 and No. 139). Of the 4 HBV carrier mothers, one (the mother of No.136) showed a positive result for the LNA-based probe real-time PCR. The G145R mutant was also undetectable in the remaining 3 mothers by deep sequencing. Moreover, deep sequencing could not detect the G145K mutant in the mother whose child was infected with the G145K as a major population. The mutation frequency by deep sequencing was 10-fold lower that than of the cloning technique and the LNA-based probe real-time PCR.

**Fig 1:**
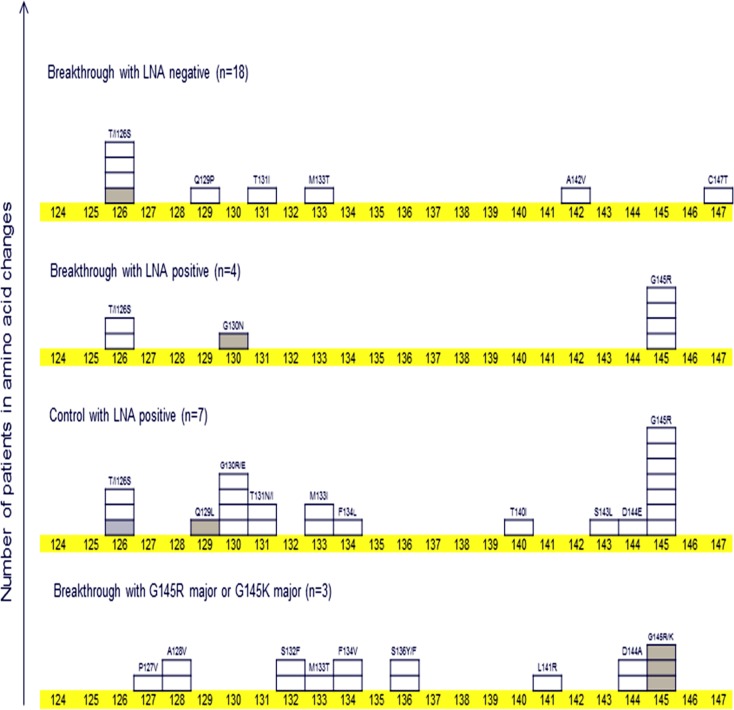
The number of patients with amino acid changes in the “a” determinant region (aa 124–147) among patients who were evaluated by deep sequencing. One box indicates one patient. A white coloured box indicates an amino acid change that exists as a minor population. A gray coloured box indicates an amino acid change that exists as a predominant population.

On the basis of the results of deep sequencing, we evaluated the frequency of amino acid changes including major and minor populations (frequency >0.4% and the number of mutation >10) in the “a” determinant region. As shown in Table 4, thirty-two patients, who were evaluated by deep sequencing, were classified into 4 groups: (A) breakthrough with LNA negative; n = 18, (B) breakthrough with LNA positive; n = 4, (C) control with LNA positive; n = 7, and (D) breakthrough with G145R major or G145K major; n = 3. The frequency of amino acid changes in the “a” determinant region per patient was 0.5, 1.8, 3.3 and 5.3 in groups A, B, C and D, respectively. The emergence of the G145R mutant showed a positive association with the amino acid mutations in the “a” determinant region. The highest frequency of amino acid changes was observed in children with the G145R/K mutant as a major population. These findings suggested that strong immunological pressure influencing the entire “a” determinant region was needed to produce the G145R/K mutant as a major population. The distribution of the amino acid mutations in the “a” determinant region is shown in Fig 1. In the breakthrough cohort, the T/I126S mutant showed the second highest frequency. However, the T/I126S mutant was undetectable in the breakthrough cohort with the G145R/K mutant as a major population. In contrast, the T/I126S mutant was detected in 4 (22%) of 18 patients who were negative for the LNA-based probe real-time PCR in the breakthrough cohort. Deep sequencing showed that the proximal mutations (amino acid position;143 and 144) of G145R were more detectable in G145R LNA-positive patients than in G145R LNA-negative patients. These findings suggested that G145R mutation tended to be accompanied with proximal mutations induced by the host immunological pressure.

**Table 4:** Frequency of amino acid changes in the a determinant region of S protein (aa124-147).

Patients		Frequency (no. of amino acid changes/ no. of patients)	
Breakthrough with LNA negative	(n = 18)	0.5	(9/18)
Breakthrough with LNA positive	(n = 4)	1.8	(7/4)
Control with LNA positive	(n = 7)	3.3	(23/7)
Breakthrough with G145R major or G145K major	(n = 3)	5.3	(16/3)

### Pregnant women

Between 2007 and 2014, 31 pregnant women (age: 21–39 years, median 32 years, HBeAg positive; n = 14, HBV DNA levels: median 4.4 log copies/mL) were enrolled in this study. Of the 31 pregnant women, 4 (12.9%) showed positive results for the LNA-based probe real-time PCR, and all of them had the G145R mutant as a minor population. The predictive factors of G145R in the pregnant women are shown in Table 5. All of the 4 pregnant women with the G145R mutant were positive for HBeAg, infected with genotype C and showed a high viral load (7.8 log copies/mL or more) before delivery. Although HBeAg-positive status showed a close association with the G145R mutant, univariate analysis revealed that there was no significant association between HBeA-positive status and detection of the G145R mutant (p = 0.07). The outcome of infants born to HBV carriers with G145R is shown in Table 6. The G145R mutant/wild HBV DNA ratio ranged from 1.5% to 60%, and the frequency of the G145R mutation evaluated by deep sequencing ranged from 1.5% to 23.8%. However, one pregnant woman dropped out of this study before delivery. Of the remaining 3 pregnant women, one had a 3 year-old child who was infected with G145R as a major form despite immunoprophylaxis (No. 136 in Table 3). Three babies were born from the 3 pregnant women, and none of them underwent Caesarean section. One month after the completion of immunoprophylaxis, 2 infants became negative for HBsAg and were positive for anti-HBs (136 mIU/mL and 925.9 mIU/mL). Moreover, they also showed negative results for the genotype-independent real-time PCR [30]. The remaining infant became negative for HBsAg and the genotype-independent real-time PCR after the first dose of HB vaccine and then dropped out of this study. These findings suggested that pre-existence of the G145R mutant as a minor strain in pregnant mothers did not always cause breakthrough infection in children.

**Table 5:** Predictive factors of G145R mutant in pregnant women with chronic HBV infection.

	All patients (n = 31)	LNA PCR for G145R		Univariate
		Positive (n = 4)	Negative (n = 27)	P value
Age, median, year	32 (21–39)	32	32	
HBV DNA levels, median, log copies/mL	4.4 (2.1–9.0)	8	4.3	0.1
HBeAg, positive (%)	14	4 (100)	10 (37)	0.07
HBV genotype				
A	1	0	1	
B	5	0	5	
C	21	4	17	
Unknown	4	0	4	

**Table 6:** HBV carrier pregnant women with the G145R mutant and their babies.

Age	HBV genotype	HBeAg	Serum HBV DNA level, log copies /mL	Deep sequencing G145R mutation frequency, % nucleotide position at 587: G →A	Quantitative PCR G145R Mutant/Wild HBV DNA ratio,%	Delivery at gestation (weeks)	One month after the 3rd shot of HB vaccine	
							HBsAg	anti-HBs levels, mIU/mL
*37	C	Pos	7.8	2	1.5	39	Neg	136
30	C	Pos	9	1.5	1.4	38	Neg	925.9
21	C	Pos	8.2	2.3	6.7	39	Neg (1mo**)	283 (1mo**)
34	C	Pos	7.8	23.8	60		Drop out	

*Failure of neonatal immunoprophylaxis occurred in the first child of the mother (the first child; failure cohort No. 136)

** The infant dropped out one mother after birth.

## Discussion

Because VEMs are rarely detected by Sanger sequencing in mothers whose children suffer breakthrough infection, there are two hypotheses that could explain the mechanism of the emergence of VEMs in MTCT [31–33]. One is that VEMs preexist in minor populations in mothers before delivery and are selected as predominant strains under the immunological pressure induced by immunoprophylaxis. The other is *de novo* mutation, which naturally occurs during the active replication of HBV involving an error-prone reverse transcription step after an infant is infected with HBV [34, 35]. A previous study from the UK showed that VEMs were detected in antenatal mothers’ serum in 2 cases of failed postnatal immunoprophylaxis using the amplification refractory mutation system (ARMS) [36]. However, this previous study was a retrospective study and neither showed the viral load of VEMs nor detected the G145R mutant. In contrast to the previous study, the current study demonstrated that immunoprophylaxis with a series of 3 doses of HB vaccine plus HBIG could prevent breakthrough infection in children born to pregnant women infected with the G145R mutant existing as a minor population. In addition to the G145R mutant, these mothers showed very high levels of serum HBV DNA (7.8 log copies/mL or more). On the basis of the results of the quantitative real-time PCR and deep sequencing, the population with the G145R mutant ranged from 1% to 60% of the total population of pregnant mothers. Therefore, the blood in the mothers contained at least 6 log copies/mL of the G145R mutant, which represents a high viral load. The maternal viral load is well known as the most significant contributor to breakthrough infection in MTCT [7, 10, 37]. Before this investigation was performed, we speculated that the HBV DNA levels of VEMs could also be one of the most influential factors for breakthrough infection. In fact, although the 3 mothers showed high HBV DNA levels of the G145R mutant, which are considered to increase the risk of breakthrough infection, the selection of the preexisting G145R mutant did not occur in their infants after birth. Therefore, this study failed to confirm the “preexisting theory”. Moreover, our retrospective evaluation of mothers whose children became HBV carriers despite immunoprophylaxis did not fully support the “preexisting theory”. The G145R mutant was detected in 13% of pregnant women in this study, and this frequency was virtually identical to that detected in the control group. In Japan, HB vaccines have not been introduced into the routine vaccination program. Therefore, the frequency of VEMs seems to have remained stable for many years. Thus, it is speculated that approximately 10% of infants born to HBV carriers have been exposed to the G145R mutant existing as a minor strain for a long time. However, the rate of immunoprophylaxis failure is 2% to 5% in Japan [38]. Taking these circumstances into consideration, the G145R mutant as a minor population does not always contribute to a breakthrough infection. It is well known that HBV is lymphotropic as well as hepatotropic. Previous studies reported that G145R mutant was frequently detected in peripheral blood leukocytes from HBsAg-positive and negative patients [39, 40]. Moreover, HBV persisting lymphatic cells contribute to occult HBV infection. Under the anti-HBs-specific humoral pressure, the compartmentalization of HBV in lymphocytes and the G145R escape mutation are advantageous for HBV to persist and survive. However, the present study did not investigate peripheral blood leukocytes frorm patients. The investigation of peripheral blood leukocytes might provide another clue to clarify the mechanism of breakthrough infection in MTCT.

To detect minor variants among the predominant wild-type strain, LNA-based probe real-time PCR was used in this study. The discrimination limit of the LNA-based probe real-time PCR was 0.5% of the entire viral population, but the lower detection limit of the real-time PCR was >1,000 copies/mL. This method is simple, commercially available and much less expensive than deep sequencing. Similarly, various special methods, such as ARMS, the combination of real-time PCR with melting curve analysis, gap ligase chain assays, limiting dilution cloning PCR, and the combination of real-time PCR with minor groove binder probes and nucleic acids, have been used for the detection of minor variants in a heterogeneous population [33, 36, 41–43]. The discrimination limits for these techniques are as follows: ARMS, as few 5%; the combination of real-time PCR with melting curve analysis, as few 5%; gap ligase chain reaction assays (gLCR), 1% to 5%; limiting dilution cloning PCR, 0.1%; and the combination of real-time PCR with minor groove binder probe and nucleic acid, 0.01%. The LAN-based probe real-time PCR is comparable with these methods in terms of the discrimination limit. Moreover, we compared the G145R mutant/wild DNA ratio quantified by the LNA-based probe real-time PCR with the G145R mutation frequency detected by deep sequencing. Deep sequencing detected the G145R mutant that was detected by the LNA-based probe real-time PCR in all but one patient. These findings suggest that both methods have the same sensitivity for the G145R mutant as the minor population among the predominant wild-type population. However, the calculated frequency of the G145R mutant was much lower in deep sequencing than in LNA-based probe real-time PCR. We do not have sufficient data to determine which method is more accurate to measure the proportion of minor and major strains. Using the gLCR assay, the ratio of the G145R mutant DNA to wild-type DNA ranged from approximately 3% to 74% in serum from various HBV carriers [42]. Although the PCR method used was different, the ratio of wild/mutant DNA in our analysis showed a similar level as this previous study. Neither the gLCR assay nor the LNA-based probe real-time PCR showed a ratio of G145R mutant DNA to wild-type DNA of less than 1%. The accuracy of the quantification depends on the ratio of the G145R mutant DNA to wild-type DNA. As the ratio reached the detection limit (wild:mutant = 200:1), the LNA-based probe real-time PCR could not accurately quantify the G145R mutant DNA level (data not shown). This finding highlights the discrepancy in the frequency of the G145R mutant between the LNA-based probe real-time PCR and deep sequencing.

Deep sequencing showed that the highest frequency of amino acid changes, including minor populations in the “a” determinant region, was observed in patients who had the G145R or G145K mutant as a predominant strain. The lowest frequency was observed in patients who did not have the G145R mutant as a minor population. These findings indicated that the emergence of the G145R mutant was accompanied by other amino acid changes in the “a” determinant region. Because the amino acid changes are considered to be a reflection of immunological pressure, this finding supports immunological pressure being necessary to induce the emergence of VEMs. Anti-HBs represent a powerful immunological factor for controlling HBV infection. Of the 3 patients who had the G145R mutant as a major population, HBsAg and anti-HBs co-existed in 2. A study from France reported that the coexistence of HBsAg and anti-HBs was associated an increase in “a” determinant variability in patients with chronic HBV infection [44]. The findings of the present study suggest that the co-existence of HBsAg and anti-HBs is a strong predictor for the emergence of VEMs as a predominant population in MTCT.

Except for the co-existence of HBsAg and anti-HBs, predictors of the presence of the G145R mutant remain unknown. The mutation of the surface gene of HBV is influenced by several factors such as age, disease progression, HBeAg status, and HBV genotype [45–48]. In the control cohort of this study, HBeAg-positive patients were prone to have the G145R mutant as a minor population. In addition to the control cohort, all 4 of the pregnant mothers who were positive for the LNA-based probe real-time PCR were also positive for HBeAg. However, these observations are inconsistent with a previous study showing that the presence of anti-HBe was positively associated with the number of naturally occurring amino acid mutations in the “a” determinant region [49]. Another study showed that the mutation rate of amino acids in the surface region of HBV in patients with a low viral load was much higher than that in patients with a high viral load [50]. Because serum alanine aminotransferase (ALT) values were normal or borderline and there was a high viral load in all of the patients who were positive for the LNA-based probe real-time PCR, they are considered to be in an immuno-tolerant phase [51]. In this phase, HBV is highly replicative but the host immune response is tolerant to HBV. Therefore, immunological pressure to induce amino acid/nucleotide mutations seems not to occur. Although previous studies have analysed viral sequences using Sanger sequencing, this method cannot detect minor populations that are less than 20% of the total population [52, 53]. Further studies are needed to confirm the association between HBeAg-positive status and the G145R mutant by sensitive detection systems or deep sequencing. A previous study detected the G145R mutant as a minor population (22.66%) using ultra-deep pyrosequencing in a pregnant woman with a high viral load (7.83 lU/mL) [54]. Moreover, treatment with lamivudine during the third trimester decreased the population of G145R mutants to 0.9% at 2 weeks after delivery (HBV DNA; 5.37 IU/mL), and breakthrough infection never occurred in the babies. Recent clinical guidelines recommended that pregnant women with a high viral load (>200,000 IU/mL or >10^6^ log copies/mL) and HBeAg should be treated with NAs to reduce the risk of immunoprophylaixs failure in MTCT [7, 10, 37, 55, 56]. If HBeAg-positive status is significantly associated with the G145R mutant in pregnant mothers, these recommendations will also be effective in reducing breakthrough infections caused by the G145R mutant.

In conclusion, the frequency of the G145R mutant as a minor as well as major strain was approximately one-fourth in children who were infected due to immunoprophylaxis failure. HBeAg-positive patients without a history of HB vaccinations, HBIG, or antiviral treatment tended to have the G145R mutant as a minor population, but HBeAg positivity did not show a significant association with the detection of the G145R mutant as a minor population. Although the G145R mutant as a minor population was detected in pregnant women who were HBeAg-positive and had a high viral load, none of the infants born to the mothers infected with the G145R mutation showed breakthrough infection. Thus, the pre-existence of the G145R mutation as a minor population in mothers does not always cause immunoprophylaixs failure after birth. However, treatment with NAs for HBV carrier pregnant women, based on an HBeAg-positive status and the level of HBV DNA, should be encouraged to reduce the risk of breakthrough infection.

## Supporting Information

S1 Fig The LNA-based probe real-time PCR (wild:mutant = 2:1). The limit to detect the G145R mutant DNA in the mixture of the excessive wild-type DNA is shown. The ratio of wild-type DNA to G145R mutant DNA is wild:mutant = 2:1. The G145R mutant-type DNA was distinguished from the wild-type DNA.
(TIF)

S2 Fig The LNA-based probe real-time PCR (wild:mutant = 20:1). The limit to detect the G145R mutant DNA in the mixture of the excessive wild-type DNA is shown. The ratio of wild-type DNA to G145R mutant DNA is wild:mutant = 20:1. The G145R mutant-type DNA was distinguished from the wild-type DNA.
(TIF)

S3 Fig The LNA-based probe real-time PCR (wild:mutant = 200:1). The limit to detect the G145R mutant DNA in the mixture of the excessive wild-type DNA is shown. The ratio of wild-type DNA to G145R mutant DNA is wild:mutant = 200:1. The G145R mutant-type DNA was distinguished from the wild-type DNA.
(TIF)

S4 Fig The LNA-based probe real-time PCR (wild:mutant = 2000:1). The limit to detect the G145R mutant DNA in the mixture of the excessive wild-type DNA is shown. The ratio of wild-type DNA to G145R mutant DNA is wild:mutant = 2,000:1. The LNA-based probe could not detect the G145R mutant in the mixture with the ratio 2,000:1.
(TIF)

## Abbreviations

HBV: hepatitis B virus
MTCT: mother-to-child-transmission
VEMs: vaccine escape mutants
HBIG: hepatitis B immunoglobulin
LNA: locked nucleic acid
NAs: nucleos(t)ide analogues
ARMS: amplification refractory mutation system
gLCR: gap ligase chain reaction assay
