# Supplementary figures and images for: Evaluation of the G145R Mutant of the Hepatitis B Virus as a Minor Strain in Mother-to-Child Transmission
Source: PLoS One. 2016 Nov 3;11(11):e0165674. doi: 10.1371/journal.pone.0165674 (PMC5094722; doi:10.1371/journal.pone.0165674)

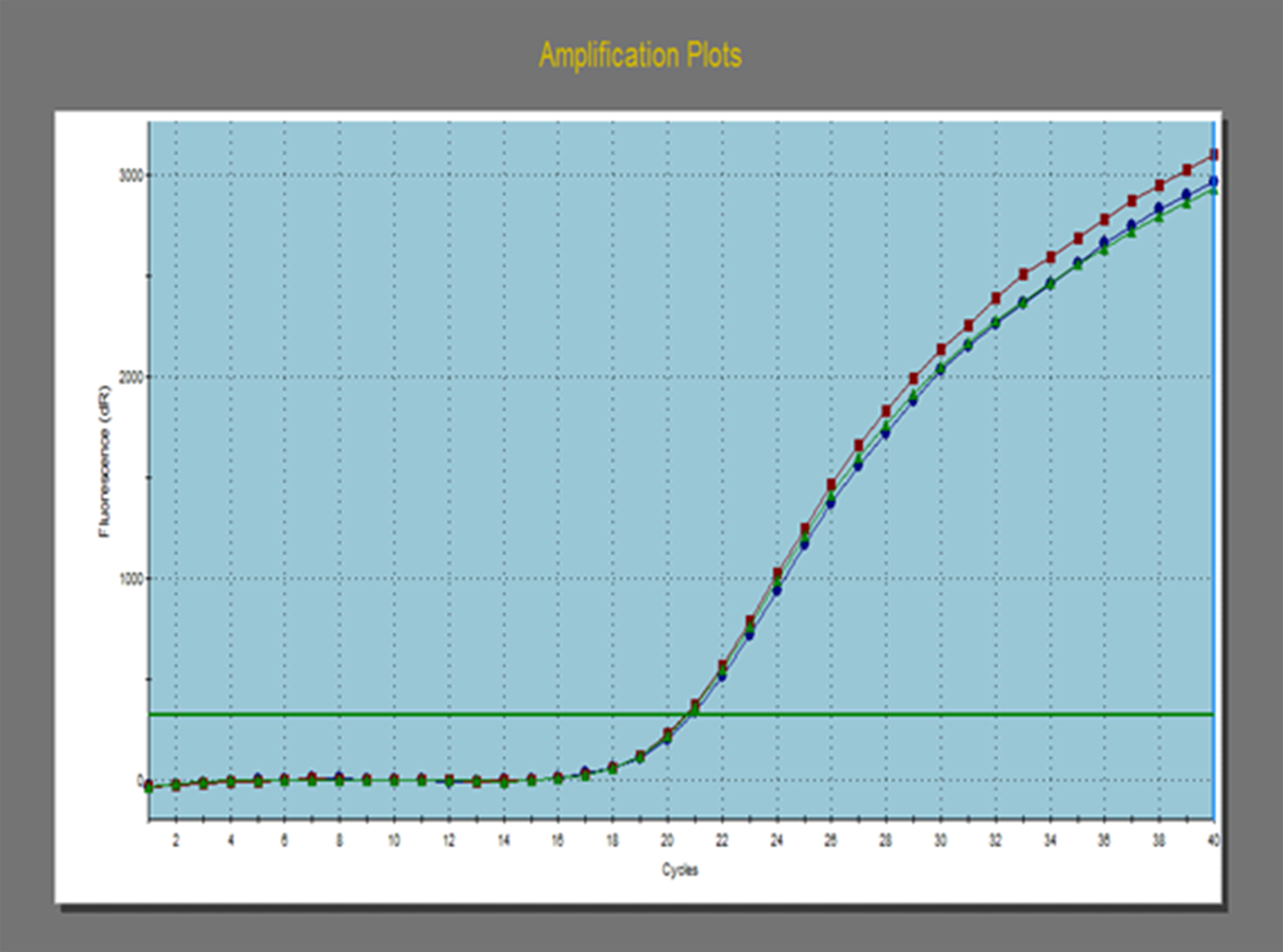

Supplement: S1 Fig — The limit to detect the G145R mutant DNA in the mixture of the excessive wild-type DNA is shown. The ratio of wild-type DNA to G145R mutant DNA is wild:mutant = 2:1. The G145R mutant-type DNA was distinguished from the wild-type DNA. (TIF) [file pone.0165674.s001.tif]

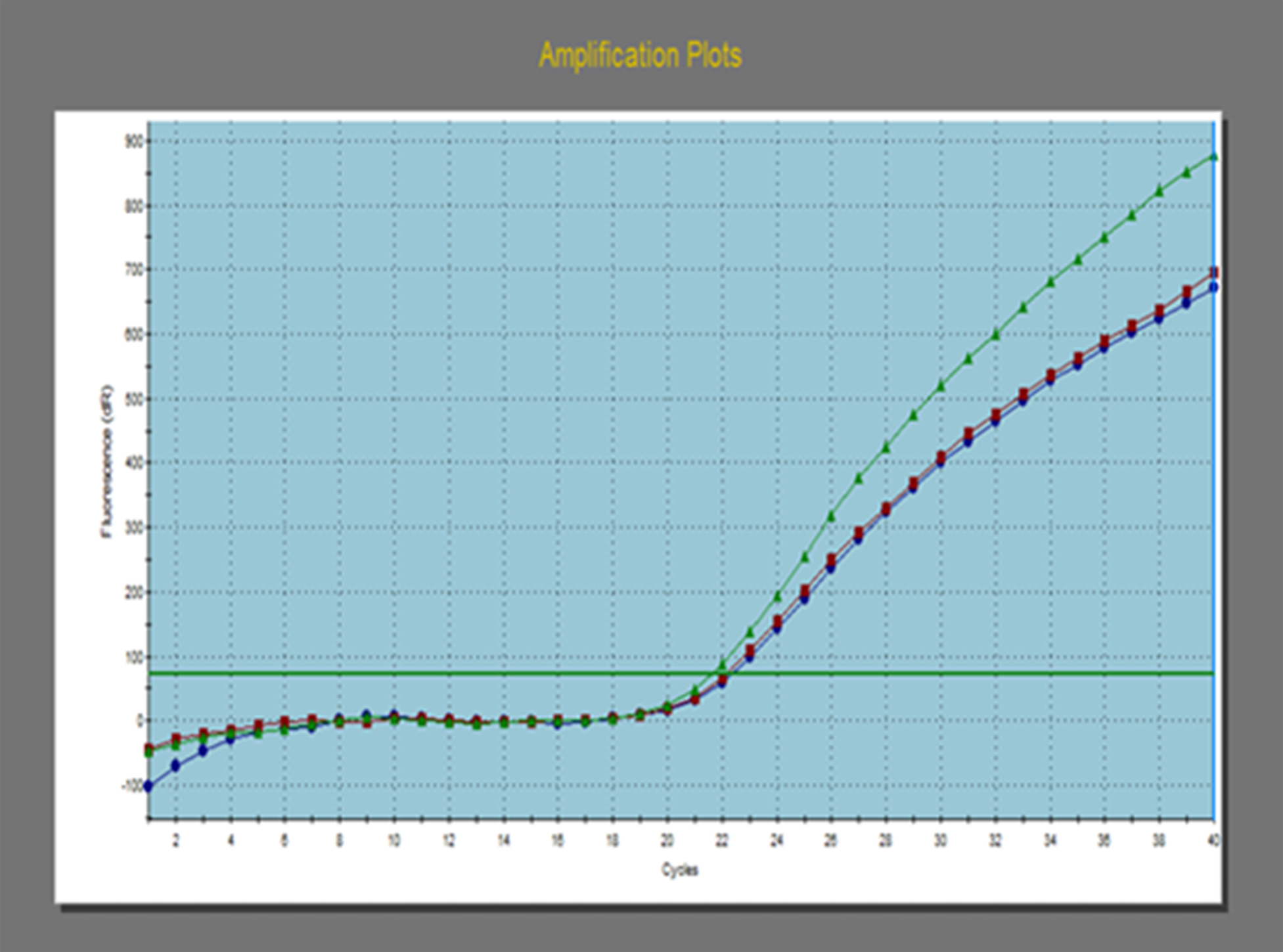

Supplement: S2 Fig — The limit to detect the G145R mutant DNA in the mixture of the excessive wild-type DNA is shown. The ratio of wild-type DNA to G145R mutant DNA is wild:mutant = 20:1. The G145R mutant-type DNA was distinguished from the wild-type DNA. (TIF) [file pone.0165674.s002.tif]

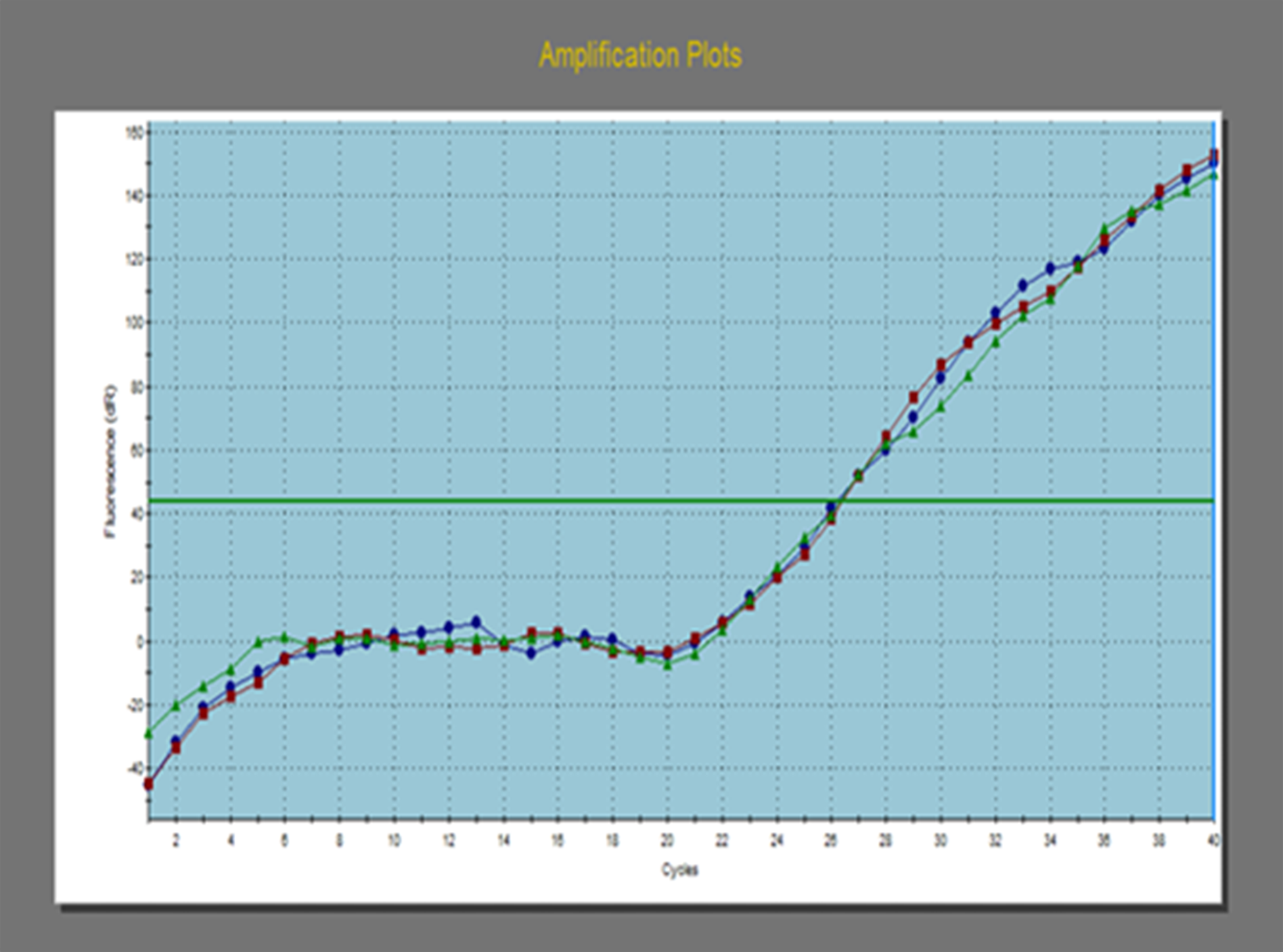

Supplement: S3 Fig — The limit to detect the G145R mutant DNA in the mixture of the excessive wild-type DNA is shown. The ratio of wild-type DNA to G145R mutant DNA is wild:mutant = 200:1. The G145R mutant-type DNA was distinguished from the wild-type DNA. (TIF) [file pone.0165674.s003.tif]

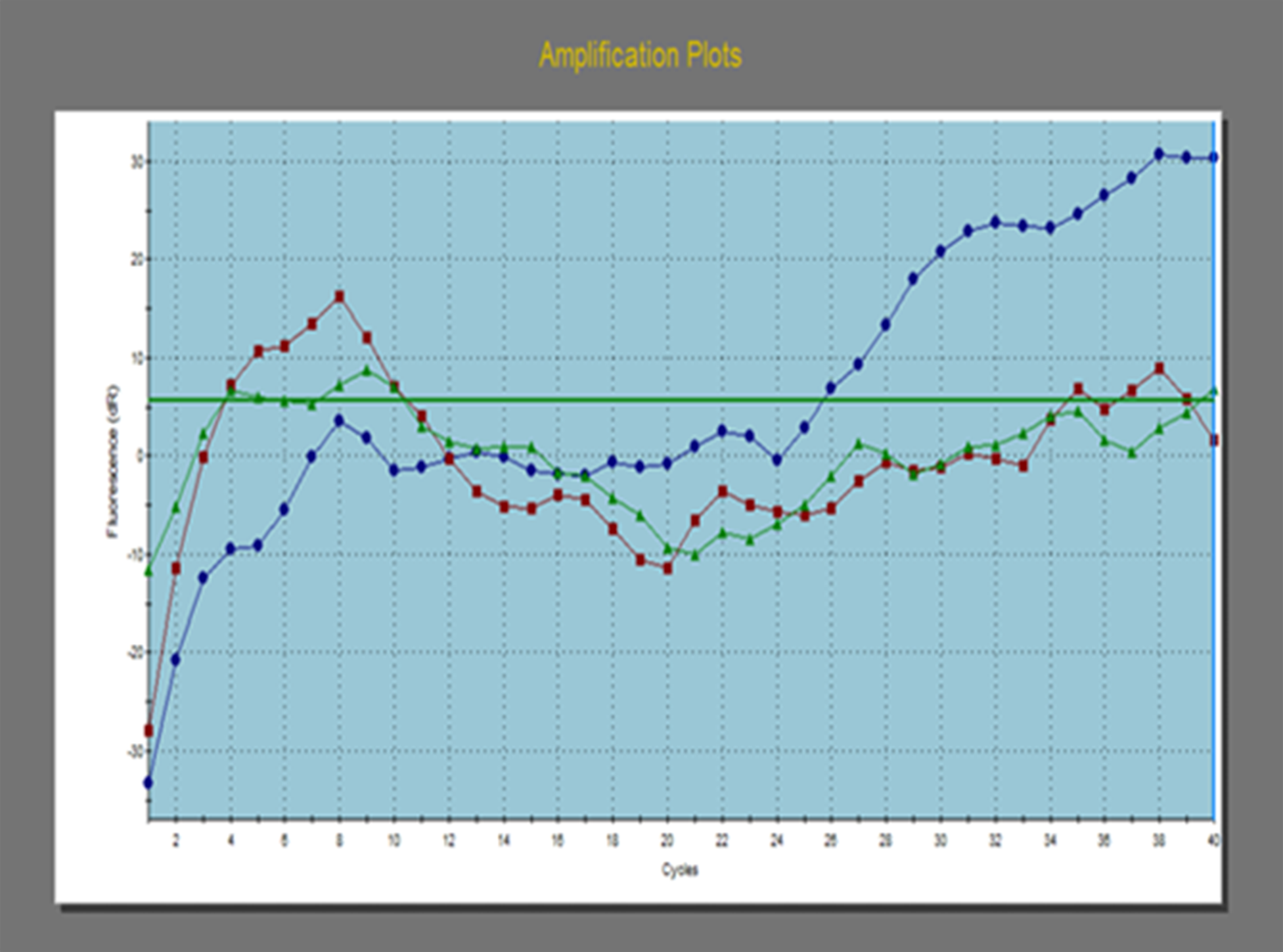

Supplement: S4 Fig — The limit to detect the G145R mutant DNA in the mixture of the excessive wild-type DNA is shown. The ratio of wild-type DNA to G145R mutant DNA is wild:mutant = 2,000:1. The LNA-based probe could not detect the G145R mutant in the mixture with the ratio 2,000:1. (TIF) [file pone.0165674.s004.tif]
